# Selenium Metabolism and Selenoproteins in Prokaryotes: A Bioinformatics Perspective

**DOI:** 10.3390/biom12070917

**Published:** 2022-06-29

**Authors:** Yan Zhang, Jiao Jin, Biyan Huang, Huimin Ying, Jie He, Liang Jiang

**Affiliations:** 1 Shenzhen Key Laboratory of Marine Bioresources and Ecology, Brain Disease and Big Data Research Institute, College of Life Sciences and Oceanography, Shenzhen University, Shenzhen 518055, China; 2014020039@email.szu.edu.cn (J.J.); 2100251007@email.szu.edu.cn (B.H.); 1900251009@email.szu.edu.cn (J.H.); jiangliang@szu.edu.cn (L.J.); 2 Shenzhen-Hong Kong Institute of Brain Science—Shenzhen Fundamental Research Institutions, Shenzhen 518055, China; 3 Affiliated Hangzhou Xixi Hospital, Zhejiang University School of Medicine, Hangzhou 310023, China; yinghuimin@139.com

**Keywords:** selenium, selenocysteine, selenoprotein, comparative genomics, bioinformatics, evolution

## Abstract

Selenium (Se) is an important trace element that mainly occurs in the form of selenocysteine in selected proteins. In prokaryotes, Se is also required for the synthesis of selenouridine and Se-containing cofactor. A large number of selenoprotein families have been identified in diverse prokaryotic organisms, most of which are thought to be involved in various redox reactions. In the last decade or two, computational prediction of selenoprotein genes and comparative genomics of Se metabolic pathways and selenoproteomes have arisen, providing new insights into the metabolism and function of Se and their evolutionary trends in bacteria and archaea. This review aims to offer an overview of recent advances in bioinformatics analysis of Se utilization in prokaryotes. We describe current computational strategies for the identification of selenoprotein genes and generate the most comprehensive list of prokaryotic selenoproteins reported to date. Furthermore, we highlight the latest research progress in comparative genomics and metagenomics of Se utilization in prokaryotes, which demonstrates the divergent and dynamic evolutionary patterns of different Se metabolic pathways, selenoprotein families, and selenoproteomes in sequenced organisms and environmental samples. Overall, bioinformatics analyses of Se utilization, function, and evolution may contribute to a systematic understanding of how this micronutrient is used in nature.

## 1. Introduction

The essential trace element, selenium (Se), plays a critical role in the growth and development of many organisms from bacteria to humans [1,2]. Although required in very small amounts, this micronutrient has been known to be involved in a variety of biological functions. It mainly occurs in the form of selenocysteine (Sec), the 21st amino acid in the genetic code, which is naturally incorporated into the active site of selenoproteins by recoding the UGA opal codon [3]. These proteins participate in several important cellular processes, such as redox homeostasis, anti-inflammatory and antiviral actions, immune responses, hormone metabolism, and reproduction [4,5,6]. The biosynthesis of Sec and its insertion into proteins involve a complex machinery that includes both common and unique components among the three domains of life [7,8]. To date, a significant number of selenoproteins have been reported in various organisms in both prokaryotes and eukaryotes, many of which were identified using reliable bioinformatics algorithms [9,10,11,12,13]. Although the functions of many selenoproteins are not clear, most of them may play pivotal roles in antioxidation and detoxification [14].

In some prokaryotes, Se is also present in 5-methylaminomethyl-2-selenouridine (mnm^5^Se^2^U, or SeU), a tRNA selenonucleoside existing at the wobble position of the anticodons of several tRNAs, and in a Se-containing cofactor (Se-cofactor) used by certain molybdoenzymes [15,16]. Although the exact functions of the two Se utilization forms are largely unknown, it has been proposed that SeU might play a significant role in improving the accuracy and efficiency of protein translation, and the Se-cofactor could support molybdenum utilization and the function of certain molybdoproteins [17,18]. In addition, because of the close chemical similarity of Se and sulfur, Se can be metabolized and utilized by sulfur assimilation pathways; however, such a nonspecific manner is not efficient and may need much larger amounts of Se due to the lack of Se-specific enzymes. Thus, only specific Se metabolic processes will be further discussed in this review.

In the recent decade, dramatic advances in high-throughput sequencing technologies have led to the generation of complete genomic sequences of numerous organisms from all three domains of life. Moreover, the development and application of new bioinformatics strategies and methods for analyzing biological information related to trace elements offers a great opportunity to acquire more in-depth knowledge of Se utilization and function in biology. To date, a variety of genome-scale computational and comparative studies on Se metabolic pathways, selenoproteins, and selenoproteome (the complete set of selenoproteins) have been carried out in various organisms (especially in prokaryotes), which could improve our understanding of how this micronutrient is used by different organisms and how the distribution and functions of selenoproteins have been shaped by evolutionary pressures.

In this review, we mainly focus on recent advances in bioinformatics and comparative genomic analyses of the metabolism and function of Se, as well as their evolutionary trends in prokaryotes to achieve a more integrated picture of Se utilization in a wide range of organisms. We also discuss recent progress in metagenomic analysis of Se usage in environmental samples, which may provide valuable information for exploring the relationship between environmental factors and the use of this element.

## 2. An Overview of Selenium Metabolism in Prokaryotes

Se occurs as inorganic species (selenate, selenite, and/or elemental Se) and in organic forms in organisms. It appears that Se utilizes the sulfur metabolic pathways, which could be taken up, in the form of selenite/selenate, by the sulfate transport system and reduced to selenide via the assimilatory sulfate reduction system [19]. It was also reported that phosphate transporters participate in selenite uptake and biotransformation in plants, yeasts, and bacteria [20,21,22]. However, a high-affinity transport system for Se has not been identified thus far.

**Figure 1:**
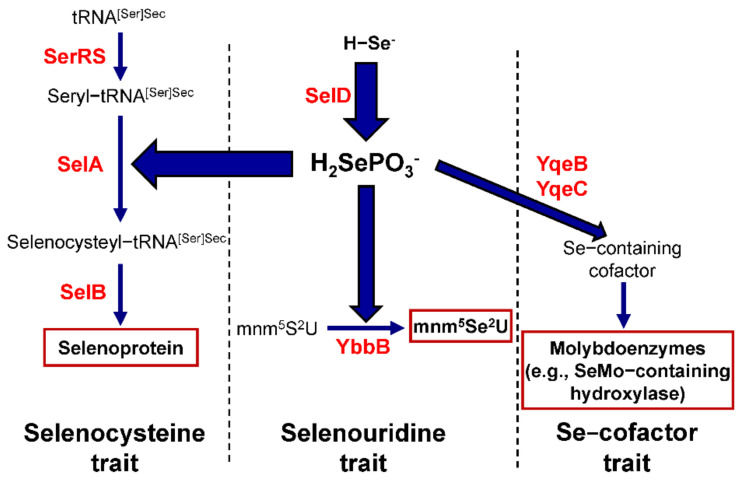
A general scheme of the three Se utilization traits in bacteria. Proteins involved in each pathway are shown in red.

In prokaryotes, the current Se metabolic pathway is comprised of three branches, the Sec, SeU, and Se-cofactor utilization traits. A general scheme of the three Se utilization traits in bacteria is shown in Figure 1. Each trait has unique genes, and selenophosphate synthetase (SelD) serves as a general signature for Se utilization.

The molecular mechanisms for the biosynthesis and incorporation of Sec into selenoproteins in prokaryotes have been comprehensively summarized in several previous reviews [23,24,25,26]. In bacteria, this process requires an in-frame UGA codon, a Sec insertion sequence (SECIS) element (a stem-loop structure located immediately 3′ of the Sec-encoding UGA codon), tRNA^Sec^ (a specific tRNA whose anticodon matches the UGA codon), and several protein factors dedicated to Sec incorporation. Briefly, the SECIS element binds to the Sec-specific elongation factor (SelB) and forms a complex with Sec-tRNA^Sec^. The tRNA^Sec^ is first charged with serine to yield seryl-tRNA^Sec^ by canonical seryl-tRNA synthetase (SerRS) and then converted to selenocysteyl-tRNA^Sec^ by Sec synthase (SelA). SelA utilizes selenophosphate as the active Se donor, which is synthesized from selenide and ATP by SelD.

In archaea and eukaryotes, although the biosynthesis of Sec adopts a similar mechanism as in bacteria, additional steps and enzymes, such as the archaeal/eukaryotic Sec synthase (SecS) and O-phosphoseryl-tRNA^Sec^ kinase (PSTK), are needed for the incorporation of Sec into protein [23,27]. However, the absence of several other eukaryotic proteins (such as SECIS-binding protein 2 and tRNA selenocysteine 1 associated protein 1) in archaea highlights the differences in Sec incorporation between archaea and eukaryotes [28]. In addition, archaeal SECIS elements are different from those in both bacteria and eukaryotes and may be localized in the 3′-untranslated region (UTR) or 5′-UTR of selenoprotein mRNAs [23,29].

With regard to the other two Se utilization traits, the 2-selenouridine synthase (YbbB, or named SelU) has been known to be responsible for the conversion of 2-thiouridine present in some bacterial tRNAs into SeU [30], while two putative gene products, YqeB and YqeC, whose functions are unclear as of yet, were predicted to be involved in the utilization of Se-cofactor [31,32]. Interestingly, only the co-existence of SelD, YqeB, and YqeC in a genome appears to be a reliable marker for the Se-cofactor trait [31].

Some other genes have also been reported to participate in Se metabolism in prokaryotes, such as cysteine (Cys) desulfurase/Sec lyase, selenate reductase, and several putative selenite reductases found in different organisms. Cysteine desulfurase/Sec lyase proteins provide sulfur derived from Cys for various processes and/or deliver Se from Sec to SelD for selenoprotein synthesis [33]. On the other hand, specific Sec lyase, which catalyzes the decomposition of Sec into alanine and selenide, was mainly detected in animals but absent in bacteria and archaea [34]. Selenate reductase is a molybdenum-dependent enzyme that is responsible for the reduction of selenate to selenite mainly in anaerobic or facultatively anaerobic organisms [35]. Selenite can be reduced to elemental Se (or selenide species) non-enzymatically by glutathione (GSH) or enzymatically by bacterial respiratory and/or detoxifying enzymes, such as periplasmic nitrite reductase and sulfite reductase [33,36,37]. In addition, several genes encoding potential selenite reductase (such as Srr from *Bacillus selenitireducens* and SerV01 from *Staphylococcus aureus*) have been proposed to be involved in this process in certain organisms [38,39,40]. It was also reported that thioredoxin (Trx) reductase is needed for selenite reduction and resistance in some bacteria, such as *Escherichia coli*, and that selenite reduction via Trx system might be an important early step for bacterial selenoprotein biosynthesis [41,42].

## 3. Computational Identification and Classification of Selenoproteins in Prokaryotes

In the past twenty years, a number of selenoprotein genes have been experimentally or computationally identified in various bacteria and archaea. Although several prokaryotic selenoprotein families, such as SelD, glutathione peroxidase (GPX), deiodinase-like (DIO), peroxiredoxin (Prx), and methionine-S-sulfoxide reductase A (MsrA), are also detected in eukaryotes, most of them occur exclusively in bacteria [43].

To date, the majority of bacterial selenoprotein genes were identified using bioinformatics approaches. Both SECIS-dependent and SECIS-independent algorithms have been developed to predict selenoprotein genes in genomic and metagenomic datasets [43,44]. The general strategy of the SECIS-based approach is to find potential SECIS elements with conserved primary and secondary structural features, then to analyze genomic context to identify the appropriate protein-coding regions, and finally, to choose good candidates for selenoprotein genes by further analysis. A program named bSECISearch was developed to predict selenoprotein genes in bacterial genomes [11]. Although a consensus structural model of bacterial SECIS elements has been suggested, putative SECIS elements in a small number of known selenoprotein genes could not satisfy the constraints for this model, implying the presence of distinct classes of SECIS elements in bacteria. On the other hand, the SECIS-independent approach uses a tblastn-based strategy to search for Cys/TGA (or Cys/Sec) pairs in the nucleotide sequence databases using a set of Cys-containing proteins, which is based on the fact that almost all selenoproteins have homologs in which Sec is replaced with Cys [12,45]. Additional criteria are further used to filter out false positives and to discover new selenoprotein genes. Using these methods, a large number of selenoproteins have been identified in both completely sequenced genomes and large-scale environmental sequencing projects.

In archaea, SECIS elements are mostly located in the 3′-UTR of selenoprotein genes and exhibit quite different structural features to those in bacteria [29]. Both SECIS-dependent and SECIS-independent methods were previously used to predict archaeal selenoprotein genes in genomic databases [12]. Compared to bacteria, only a few selenoprotein families have been identified in a limited number of archaea (Methanococcales and Methanopyrales), most of which are methanogens [46]. Recently, it was reported that the archaeon Lokiarchaeota (belonging to the Asgard superphylum) has several selenoprotein genes possessing eukaryotic-like SECIS elements, suggesting that Lokiarchaeota might be an intermediate form between the archaeal and eukaryotic Sec-encoding systems [47]. In addition, despite that no known selenoprotein could be detected in Thorarchaeota (another phylum within the Asgard superphylum), the presence of several key genes involved in selenoprotein biosynthesis indicates that Thorarchaeota may have currently unknown selenoproteins [48].

To date, more than 80 selenoprotein families and subfamilies are known in prokaryotes. The majority of these selenoproteins contain a Trx-like fold with a redox-active motif. Although more and more selenoprotein genes have been identified in different genomic and metagenomic datasets, a complete collection of prokaryotic selenoproteins is still lacking. Here, we have summarized all the previously reported selenoproteins (including both experimentally verified and in silico predicted) from the literature [11,12,45,49,50,51,52,53,54,55,56,57,58,59,60,61,62,63,64,65,66,67,68] and generated the most comprehensive list of selenoproteins in prokaryotes thus far (Table 1). A total of 87 selenoprotein families or subfamilies are included. The naming of selenoproteins in this review is mainly based on the conserved domains detected in their protein sequences, which may provide uniformity to the designation of these proteins. In addition, if two selenoproteins contain the same domain but different Sec sites or Sec-related motifs, they are considered as different subfamilies, such as Prx-like thiol:disulfide oxidoreductase (pfam00578, UxxC/UxxU, x represents any amino acid) and UGC-containing Prx-like protein (pfam00578, UGC), as well as rhodanese-related sulfurtransferase COG0607 form 1 (COG0607, no motif) and rhodanese-related sulfurtransferase COG0607 form 2 (COG0607, CxU). As mentioned above, most of these selenoproteins were predicted by bioinformatics methods and their functions are not clear. However, considering that almost all selenoproteins whose functions are known play important roles in antioxidant defense and that most of the predicted selenoproteins are homologous to diverse thiol-based oxidoreductases, it is very likely that the majority of these uncharacterized selenoproteins serve redox functions.

Since tRNA^Sec^ is a key component for selenoprotein biosynthesis, its efficient identification would be beneficial to the prediction of new selenoprotein families if no known selenoproteins could be detected in genomes with tRNA^Sec^. A tRNA^Sec^-specific identification tool named Secmarker was recently developed based on conserved structural features of those tRNAs, which revealed new insights into the biology of tRNA^Sec^ and led to the discovery of novel bacterial selenoprotein families [68].

## 4. Comparative Genomics of Selenium Utilization in Prokaryotes

Comparative genomics is an important research field in bioinformatics, which provides a powerful strategy for unraveling the functions and evolutionary dynamics of various genes, pathways, and other characteristics conserved or unique across different species or lineages [69,70]. By using comparative genomic approaches in the field of trace elements, we may better understand trace element-dependent cellular processes and proteins that an organism has [71,72]. To date, several comparative genomic studies have analyzed the distribution and evolutionary trends of Se metabolic pathways and/or selenoproteins in a variety of bacteria and archaea, which allow for a general understanding of the status of Se metabolism and function in the two kingdoms [46,47,65,73,74,75,76,77,78].

An early study examined the Sec biosynthetic pathway and known selenoproteins in approximately 600 bacterial and archaeal genomes [46]. Sec was found to be utilized by very few archaea (Methanococcales and Methanopyrales) and approximately one-fourth of sequenced bacteria belonging to Deltaproteobacteria, Epsilonproteobacteria, and many other phyla, whereas only a small number of bacterial lineages (such as Cyanobacteria and Mollicutes) appeared to lack the ability to use this uncommon amino acid. This may imply that Sec utilization is an ancient trait that was once common to the majority of organisms in bacteria but has been selectively preserved or adopted in proteins and organisms during evolution. The majority of selenoprotein-rich organisms were anaerobic organisms in Deltaproteobacteria and Clostridia, including a syntrophic propionate-oxidizing deltaproteobacterium *Syntrophobacter fumaroxidans* that has the largest prokaryotic selenoproteome reported so far (39 selenoprotein genes). Although the reasons for such an unusual distribution of Sec utilization are not clear, a dynamic and delicate balance between Sec acquisition and selenoprotein loss events observed in different phyla may partially explain the discrepancy between the catalytic advantages provided by Sec and its restricted use in nature [46].

Several recent comparative studies have analyzed different Se metabolic pathways, related key genes, and selenoproteomes by using much more sequenced prokaryotic genomes, which not only suggest new functions for several known Se metabolic genes but also imply novel genes involved in Se metabolism and homeostasis. For example, one study reported the presence of a SelD-like protein in certain orders of Crenarchaeota (such as Sulfolobales and Thermoproteales), which has originated from SelD (the key gene essential for all known Se utilization traits) and might be involved in sulfur metabolism (for example, the biosynthesis of a certain thiophosphate compound) in hyperthermophilic sulfur-reducing archaea [73]. Another study traced the evolutionary history of SelD (or SPS) genes in both prokaryotes and eukaryotes and revealed different fusions between SelD and other genes as well as independent gene duplications and associated subfunctionalization events, indicating a particular “functional evolution path” of SelD genes [74]. Lin et al. explored the distribution of known Se metabolic genes in more than 2300 bacterial and archaeal genomes and proposed a new model for Se homeostasis in bacteria [75]. Based on the sequence and phylogenetic analyses of their neighboring genes, several new gene products were predicted to be involved in Se metabolism, including YedE (a possible Se-related transporter), YedF (a protein involved in Se-related redox processes), DUF3343-containing protein (a possible chaperon involved in Se trafficking), and LysR_Se (a Se-specific transcriptional regulator), which might be useful for a further understanding of the mechanism underlying the metabolism and homeostasis of Se in prokaryotes. Some of these genes, such as LysR_Se (or named HrsM), have been later experimentally verified [76].

**Figure 2:**
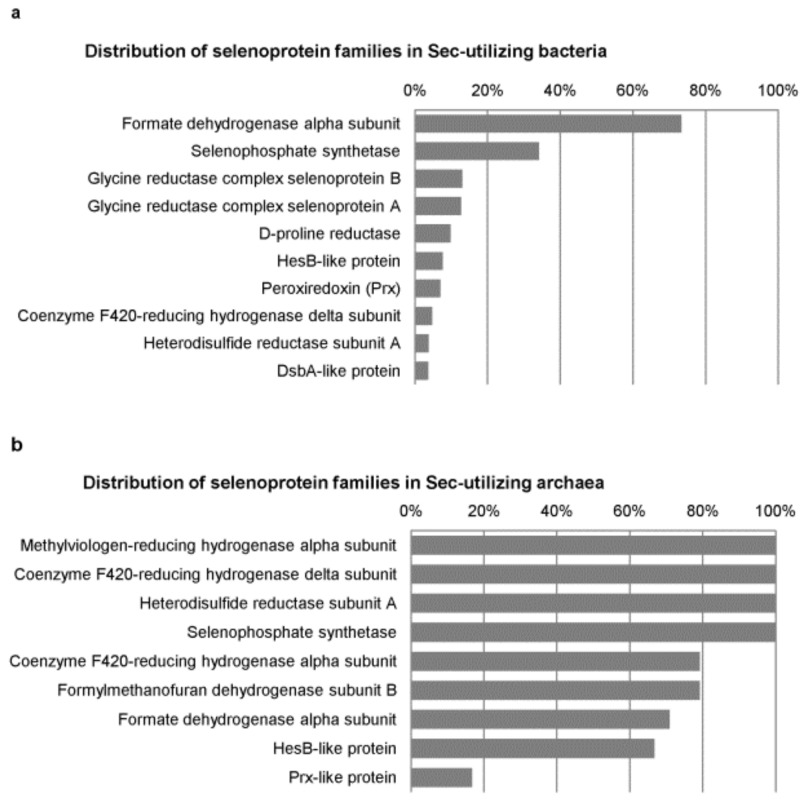
Distribution of the top ten selenoprotein families in Sec-utilizing prokaryotes. (**a**) Bacteria; (**b**) archaea. Data used to generate this figure can be found in refs. [46,47,65].

A more extensive investigation of the distribution and evolution of Se metabolic pathways and selenoproteins in bacteria have been conducted by analyzing more than 5200 genomes, which demonstrates the largest picture of Se utilization in this kingdom [65]. Although only one third of sequenced bacteria had at least one Se utilization trait, significant overlaps exist between different traits, suggesting that the occurrence of one Se trait may be beneficial to acquisition of others, probably partially due to the presence of SelD. Interestingly, SelD orthologs were also found in some organisms that do not have any of the known Se traits, implying the presence of an unknown Se utilization trait. Several genes (such as isochorismatase-like protein and ABC transporter-related ATPase) were predicted to be associated with this novel SelD-based Se utilization trait. Among all known selenoproteins, formate dehydrogenase alpha subunit (FdhA), SelD, glycine reductase complex selenoprotein B (GrdB), glycine reductase complex selenoprotein A (GrdA), and D-proline reductase (PrdB) were the five most widespread bacterial selenoprotein families (Figure 2a). Besides Deltaproteobacteria and Clostridia, Synergistetes was also considered as a selenoprotein-rich phylum (the majority of sequenced organisms were selenoprotein-rich organisms). The Sec and Se-cofactor traits appeared to favor host-associated conditions, whereas the SeU trait preferred aquatic environments. In addition, low oxygen or anaerobic conditions might be associated with the Se-cofactor trait and the evolution of new selenoprotein genes. It is possible that, under normal oxygen conditions, organisms could not tolerate the highly reactive Sec residue, which could be easily oxidized and then support the production of reactive oxygen species.

In addition, the complete loss of Sec biosynthesis machinery and selenoproteins was recently found to have occurred in closely related species or even different strains of the same species. Cravedi et al. analyzed the evolution of Sec biosynthesis machinery genes and the selenoproteome of several *Helicobacter pylori* strains and related Epsilonproteobacteria, which revealed that Sec incorporation system was lost prior to the split of *H. acinonychis* and *H. pylori*, probably due to the adaptation of their progenitor to the host [67]. Miller et al. analyzed a variety of Campylobacter species and found that all sequenced *C. lanienae* genomes have completely lost Sec biosynthetic genes and selenoprotein genes, which is a unique feature of this newly defined clade [77]. Similarly, compared to other *C. jejuni* strains, deletion of the genes encoding Sec insertion machinery and selenoproteins was detected in two *C. jejuni* strains isolated from guinea pigs, which might be associated with host specialization related to guinea pig diet (e.g., a low Se dietary requirement with poor Se dietary reserve), anatomy, and physiology [78].

In archaea, only nine selenoprotein families were previously discovered in a small number of organisms in Methanococcales, Methanopyrales, and Lokiarchaeota [27,46,47]. Among them, methylviologen-reducing hydrogenase alpha subunit (MvhA/VhuU), coenzyme F420-reducing hydrogenase delta subunit (FrhD/MvhD/VhuD), heterodisulfide reductase subunit A (HdrA), and SelD were detected in all Sec-utilizing archaea (Figure 2b). As most archaeal selenoproteins are involved in methanogenesis, Se-free isoforms (Cys-containing homologs) of these selenoproteins are present in a much wider range of organisms that do not use Sec [27]. The reported archaeal selenoproteomes had a relatively narrow distribution (7~12 selenoproteins), and Lokiarchaeota appeared to have the largest archaeal selenoproteome known so far (12 selenoprotein genes) [47]. With the increasing number of sequenced archaeal genomes, it is possible to identify additional selenoprotein families in this kingdom. Anyway, these findings should be helpful for a further understanding of the macro-evolutionary dynamics of Se metabolism and function in nature.

Very recently, a bioinformatics study examined SelB sequences in certain Alphaproteobacteria and found that the tRNA^Sec^ is completely encoded inside the C-terminal extended *selB* gene in diverse species of Alphaproteobacteria (such as Rhodobacterales, Rhodospirillales, and Caulobacterales) [79]. This is the first example of an entire tRNA sequence nested in the coding region of mRNA in bacteria. Similar overlapping traits were also detected in Gammaproteobacteria and Nitrospirae, which may indicate a new approach for maintaining homeostasis between SelB and Sec-tRNA^Sec^ and for controlling the expression level of *selB* in bacteria.

## 5. Comparative Metagenomics of Selenium Utilization

Metagenomic analysis has become a very popular tool for understanding the microbial diversity and their metabolic potentials in different environments. In the past decade, the rapidly expanding field of metagenomics has produced a vast amount of environmental genomic data, from the world’s oceans to human gut microbiota [80,81,82]. Previous analyses of the relationship between living conditions and Se utilization in sequenced prokaryotes have revealed that certain environmental factors can affect not only the distribution of different selenoprotein families but also the selenoproteomes [46,65]; however, so far, very limited studies have been performed to explore the utilization and function of Se in microbial communities, almost all of which have focused on marine environments.

The oceans contain a large number of microbes that cannot be cultivated in vitro. It has been reported that marine biogeochemical cycles and the utilization of Se have co-evolved and could influence each other [83,84]. By using the sequence and other data from large-scale marine metagenomics projects, such as the Global Ocean Sampling (GOS) expedition (one of the largest and geographically most comprehensive metagenomic datasets), several studies have investigated the occurrence and evolution of Se metabolism pathways and selenoproteins, which provide a basis for the utilization and roles of this micronutrient in global marine microbial communities.

An early comparative analysis of Se utilization in the marine microbes was conducted based on 44 diverse aquatic samples from the GOS dataset, which generated the first map demonstrating the distribution and evolution of Sec and SeU utilization traits in a global biogeographical context [64]. Approximately 60 prokaryotic selenoprotein families could be detected, and selenoprotein W(SELENOW)-like and SelD were the most abundant selenoprotein families in these samples. Higher water temperature and marine environments were found to be associated with the increased use of Sec. On the other hand, the SeU utilization trait showed a relatively independent relationship with the Sec trait. No significant correlation could be found between SeU utilization and marine habitat types or geographic location. Thus, although both Se traits require Se supply and might influence each other, additional factors may play more important and specific roles in the evolution of individual Se utilization traits.

A much larger comparative metagenomic study was recently performed to examine the biogeographic distribution of both selenoprotein genes and metalloprotein genes in a diverse range of marine, freshwater, and hypersaline environments from the updated GOS dataset [85]. More than 4300 selenoprotein genes corresponding to 59 previously described selenoprotein families were predicted, becoming the largest dataset of marine selenoprotein genes reported to date. The prominent selenoproteins include SELENOW-like, alkylhydroperoxidase(AhpD)-like, SelD, UGSC-containing proteins, Prx, and several other Prx- and Trx-like proteins. A number of selenoprotein-rich and selenoprotein-poor samples were identified, suggesting an active or inactive usage of this element in various marine sites. Besides water temperature, several environmental factors (such as sample depth, ocean acidification, and concentrations of silicate/nitrate/phosphate) might also contribute to the evolution of different selenoprotein genes in the marine microbial world. Moreover, significant positive correlations between Se utilization and that of some trace metals (such as nickel and molybdenum) were found, implying that certain factors could simultaneously activate or inhibit the use of multiple elements in marine microbes. This may provide new clues for a better understanding of the relationship between the utilization of these elements in marine environments.

In addition to the GOS project, the Tara Oceans metagenomic dataset was also used to characterize Se utilization in various marine microbial communities [86]. By identifying the genes involved in different Se utilization traits in marine samples collected from oceans around the world, several regions with samples rich or poor in Se utilization traits were identified. Moreover, a higher water temperature and mesopelagic zone of water depth appeared to be favorable for Se utilization, which provides useful information for the general features of Se utilization in ocean samples.

Except sea water, the use of Sec in marine sediment microbiome was also investigated based on the metagenomic data from the sediments of a deep-ocean industrial waste dump site [87]. By analyzing the reconstructed genomes of Deltaproteobacteria, which are the most abundant mat organisms in the sediments, more than 30 putative selenoprotein genes (including both previously reported and newly predicted) were found, indicating a highly active utilization of Sec in the dominant deltaproteobacteria in marine sediments. Although the majority of these proteins are redox-related proteins, the presence of Sec in multiple non-redox proteins implies additional, as of yet unknown, roles of Se. Further analysis indicated a wide geographic distribution of similar groups of specialized Deltaproteobacteria in various environments, such as sulfidic sites and terrestrial/estuarine environments. These findings may suggest an important biogeochemical role for those specialized Deltaproteobacteria in the process of Se cycle in the ocean. 

To date, metagenomic analyses of Se metabolism and selenoproteins in other environments are very rare. Two recent metagenomic studies examined the abundance of selenate reductase genes in different biosamples from coalmine-impacted aquatic sediments and membrane biofilm reactors, which revealed that nitrate and sulfate could inhibit selenate reduction (a part of the metabolism for synthesis of selenoproteins) and further influence Se status and/or selenoprotein biosynthesis [88,89]. Future efforts are needed to investigate the evolutionary trends of Se utilization in other types of environments.

## 6. Conclusions

Bioinformatics provides a powerful tool for investigating Se utilization, function, and evolution in different kingdoms of life. Most of these studies have aimed for the identification of selenoprotein genes in different genomic datasets. Compared to other trace elements, such as metals, prediction of selenoprotein genes and the selenoproteome in different organisms is easier and more reliable, due to several highly specific sequence-structural features for Sec insertion machinery. More than 80 selenoprotein families or subfamilies have been reported in the recent decade, most of which are thiol-based oxidoreductases. Recent progress in comparative genomic research of Se metabolism and selenoproteins in prokaryotes has provided important information about the general principles of Se utilization and evolutionary trends in biology. In addition, comparative metagenomics may offer new insights into the use of Se in a much wider range of microbes, as well as its relationship to various environmental conditions. In the future, with the rapid increase in the number of sequenced genomes and improved computational techniques for identifying more selenoprotein genes, bioinformatics and comparative genomics/metagenomics will play a more important role in elucidating Se utilization and function in nature.

## Figures and Tables

**Table 1:** A complete list of currently reported selenoprotein families/subfamilies in prokaryotes.

Selenoprotein Family or Subfamily Name	Domain ID (Name)	Sec-Related Motif	Representative Sequence (Genbank/Refseq)	Ref.
**Experimentally verified (16)**				
Formate dehydrogenase alpha subunit *	COG0243 (BisC)	-	WP_010904702.1	[49]
Formylmethanofuran dehydrogenase subunit B *	COG1029 (FwdB)	-	CAA67419.1	[50]
Selenophosphate synthetase *	COG0709 (SelD)	UxxK	WP_083774555.1	[51]
Coenzyme F420-reducing hydrogenase alpha subunit *	COG3259 (FrhA)	UxxC	WP_083774535.1	[52]
Methylviologen-reducing (or F420-nonreducing) hydrogenase alpha subunit *	COG3259 (FrhA)	UxxC	P0C1V6.2	[27]
Coenzyme F420-reducing hydrogenase delta subunit *	COG1908 (FrhD)	-	WP_010870703.1	[53]
Heterodisulfide reductase alpha subunit *	COG1148 (HdrA)	CxxU	WP_162484757.1	[54]
HesB-like protein *	TIGR01911 (HesB_rel_seleno)	-	WP_083774540.1	[55]
Glycine reductase complex selenoprotein A	pfam04723 (GRDA)	CxxU	WP_079747582.1	[56]
Glycine reductase complex selenoprotein B	pfam07355 (GRDB)	UxxC	WP_246895825.1	[56]
D-proline reductase	TIGR04483 (D_pro_red_PrdB)	UxxC	WP_079281142.1	[57]
Peroxiredoxin (Prx)	COG1225 (Bcp)	TxxU	WP_011365628.1	[58]
Thioredoxin (Trx)	pfam00085 (Thioredoxin)	UxxC	WP_010956703.1	[59]
Glutaredoxin (Grx)	pfam00462 (Glutaredoxin)	UxxC	WP_010943784.1	[60]
Methione sulfoxide reductase A	COG0225 (MsrA)	-	MBI4965933.1	[61]
Arsenite methyltransferase	PRK11873 (arsM)	-	WP_011987699.1	[62]
**Predicted (71)**				
Radical SAM domain protein	TIGR04167 (rSAM_SeCys)	-	AAR34688.1	[11]
Rhodanese-like domain-containing protein	pfam00581 (Rhodanese)	-	WP_010941598.1	[11]
Rhodanese-related sulfurtransferase COG0607 form 1	COG0607 (PspE)	-	MBM9537886.1	[11]
Rhodanese-related sulfurtransferase COG0607 form 2	COG0607 (PspE)	CxU	TKB26178.1	[11]
Prx-like thiol:disulfide oxidoreductase *	pfam00578 (AhpC-TSA)	UxxC, UxxU	WP_010940744.1	[12]
Thiol:disulfide interchange protein	pfam13098 (Thioredoxin_2)	UxxC	WP_011366075.1	[12]
Selenoprotein W (SELENOW)-like protein	pfam10262 (Rdx)	CxxU	AOH51717.1	[12]
Glutathione peroxidase (GPX)-like protein	pfam00255 (GSHPx)	UxxT	WP_010957027.1	[12]
Homolog of AhpF N-terminal domain (Grx-like domain protein)	TIGR02187 (GlrX_arch)	UxxC	ABB15282.1	[12]
DsbG-like protein	pfam13462 (Thioredoxin_4)	UxxC	*WP_012258530.1* **	[12]
Fe-S oxidoreductase-like protein	COG0247 (GlpC)	-	WP_174406253.1	[12]
DsrE-like protein	pfam02635 (DsrE)	UxxC	WP_014524487.1	[12]
FAD-dependent oxidoreductase (CoA-disulfide reductase, NADH oxidase)	COG0446 (FadH2)	-	WP_011365774.1	[12]
Distant Alkylhydroperoxidase (AhpD) homolog	COG0599 (YurZ)	CxxU	*AAR36519.2*	[12]
AhpD-like protein	COG2128 (YciW)	CxxU	MCB9421940.1	[45]
Arsenate reductase	COG1393 (ArsC)	UxxS	MBT3519430.1	[45]
Molybdopterin-synthase adenylyltransferase MoeB	COG0476 (ThiF)	-	MBT7809913.1	[45]
DsbA-like protein	pfam01323 (DSBA)	UxxC	NIP15863.1	[45]
Glutathione S-transferase-like (GST-like)	COG0625 (GstA)	-	*PPR62222.1*	[45]
Deiodinase-like protein	pfam00837 (T4_deiodinase)	UxxC	*MBO99264.1*	[45]
Thiol-disulfide isomerase-like protein	pfam13905 (Thioredoxin_8)	UxxC	*MAK15852.1*	[45]
Carboxymuconolactone decarboxylase(CMD)-like protein	pfam02627 (CMD)	CxxU	MBW1767730.1	[45]
Hypothetical protein 1 (Sargasso Sea metagenome)	-	CxxU	*MBR86424.1*	[45]
OsmC-like protein	COG1765 (YhfA)	UxxT	*MBR72571.1*	[45]
Rhodanase-related sulfurtransferase	COG2897 (SseA)	-	*MQG53192.1*	[45]
NADH:ubiquinone oxidoreductase subunit E	COG2209 (NqrE)	TxxU	-	[45]
Putative mercuric transport protein	pfam02411 (MerT)	-	ABB16073.1	[63]
Cation-transporting ATPase, E1-E2 family	COG2217 (ZntA)	UxxC	ABB15669.1	[63]
Methylated-DNA-protein-cysteine methyltransferase	COG0350 (AdaB)	-	ABB14497.1	[63]
UGSC-containing protein	-	UxxC	ABI76733.1	[63]
DUF3179 domain-containing protein	pfam11376 (DUF3179)	UxxC/T	*MBW1804167.1*	[63]
YHS domain-containing protein	pfam04945 (YHS)	-	-	[63]
Putative redox protein	-	-	*KGM38912.1*	[63]
DUF166 domain-containing protein	pfam02593 (DUF166)	-	-	[63]
DUF1573 domain-containing protein	pfam07610 (DUF1573)	UGC	*CAB1076174.1*	[63]
Hypothetical protein OS_HP3	-	-	SMF39960.1	[63]
Putative mercuric reductase	PRK13748 (PRK13748)	UxxU	*CAB1070815.1*	[63]
Hypothetical protein OS_HP4	-	UxxC	-	[63]
Cobalamin synthesis protein CobW-like	COG0523 (YejR)	UxxC	*CAB1077436.1*	[63]
AhpC/TSA family protein	pfam13911 (AhpC-TSA_2)	UxxS	*CAB1081847.1*	[63]
Hypothetical protein OS_HP5	-	-	-	[63]
Distant Grx-like protein 1	TIGR02196 (GlrX_YruB)	UxxT	MBW2590879.1	[64]
Arsenate reductase-like protein	COG1393 (ArsC)	UxxC	*MAM02162.1*	[64]
Fe-S cluster domain-containing protein	PRK07118 (PRK07118)	UxxC	*ABC78902.1*	[64]
(2Fe-2S)-binding protein (copper chaperone Copz family) form 1	cd10141 (CopZ-like_Fer2_BFD-like)	-	WP_245779778.1	[64]
(2Fe-2S)-binding protein (copper chaperone Copz family) form 2	cd10141 (CopZ-like_Fer2_BFD-like)	-	MBF1269327.1	[64]
Hypothetical protein predicted in Moorella thermoacetica	-	-	*WP_155767724.1*	[64,65]
Alkylmercury lyase MerB-like protein	pfam03243 (MerB)	-	WP_238493467.1	[64,65]
DUF1858 domain-containing protein	pfam08984 (DUF1858)	CxxU	WP_012065717.1	[64,65]
Proline reductase-associated electron transfer protein PrdC form 1	TIGR04481 (PR_assoc_PrdC)	CxxU	WP_243183503.1	[64,66]
Proline reductase-associated electron transfer protein PrdC form 2	TIGR04481 (PR_assoc_PrdC)	-	WP_245122565.1	[64,65]
cytochrome c family protein	pfam13435 (Cytochrome_C554)	-	*WP_013164579.1*	[64,65]
MtrB/PioB family outer membrane beta-barrel protein	pfam11854 (MtrB_PioB)	-	*WP_005997773.1*	[64]
UshA-like protein	COG0737 (UshA)	CxU	*WP_013162925.1*	[64]
C-GCAxxG-C-C family protein	pfam09719 (C_GCAxxG_C_C)	-	WP_012158890.1	[64]
CO dehydrogenase/acetyl-CoA synthase gamma subunit	COG1456 (CdhE)	-	*WP_012647565.1*	[64]
YeeE/YedE family protein	pfam04143 (Sulf_transp)	-	WP_012471001.1	[64,65]
UGC-containing Prx-like protein	pfam00578 (AhpC-TSA)	UGC	*MBL6689828.1*	[64]
Ferredoxin-thioredoxin reductase	COG4802 (FtrB)	CxU	*MBG54406.1*	[64]
Trypsin-like serine protease	pfam00089 (Trypsin)	-	-	[64]
Putative regulatory protein, FmdB family	TIGR02605 (CxxC_CxxC_SSSS)	U/CxxU	-	[64]
PDZ domain-containing protein	pfam13899 (Thioredoxin_7)	CxxU	*MBM3766709.1*	[64]
Hypothetical protein GOS_A	-	-	-	[64]
Hypothetical protein GOS_B	-	-	NBR19009.1	[64]
Hypothetical protein GOS_C	cd02973 (TRX_GRX_like)	UxxC	*MBI79719.1*	[64]
Redoxin family protein	-	UxxC	*MBA3499694.1*	[64,65]
Crotonase/enoyl-CoA hydratase family protein	PRK06023 (PRK06023)	-	*KAA1296466.1*	[65]
Cobalamin binding protein BtuF	cd01144 (BtuF)	CxxU	*RUA21600.1*	[65]
KCU-star family selenoprotein (or DUF466 protein)	NF033934 (KCU-star)	-	*WP_052061029.1*	[67]
Thioredoxin-like selenoprotein Sec.1	pfam13192 (Thioredoxin_3)	CxU	WP_232817751.1	[68]
Thioredoxin-like selenoprotein Sec.2	pfam13192 (Thioredoxin_3)	UxC	WP_218069652.1	[68]

* Selenoprotein families detected in both archaea and bacteria. ** Italic font: only truncated form of selenoprotein is annotated (no Sec included).

## Data Availability

Not applicable.
